# Seborrheic keratoses and severe hypoinsulinemic hypoglycemia associated with insulin grow factor 2 secretion by a malignant solitary fibrous tumor

**DOI:** 10.1186/s13098-016-0148-2

**Published:** 2016-04-29

**Authors:** Andreia Latanza Gomes Mathez, Debora Moroto, Sergio Atala Dib, Joao Roberto de Sa

**Affiliations:** Endocrinology Division, Escola Paulista de Medicina, Federal University of São Paulo, São Paulo, SP Brazil

**Keywords:** Insulin-like growth factor 2, Hypoglycemia, Tumor, Seborrheic keratosis

## Abstract

A rare sign of some malignant tumors is a sudden eruption of multiple seborrheic keratoses called Leser-Trélat sign. Overproduction of insulin-like growth factor-2 (IGF2) or its precursor is the main mechanism related to non-islet cell tumor hypoglycemia. Doege-Potter syndrome is the name given to paraneoplastic hypoinsulinemic hypoglycemia in presence of a solitary fibrous tumor. This report describes a case of a patient with hypoinsulinemic hypoglycemia and Leser-Trélat sign associated with a malignant solitary fibrous tumor with IGF2 secretion. Both conditions have improved after tumor excision.

## Background

Cutaneous morphology can be modified by endocrine and metabolic diseases and skin lesions might serve as a window to the early diagnosis and treatment of various hormone-secreting tumors [1]. An example is a sudden eruption of multiple seborrheic keratosis called as Leser-Trélat sign and it is more associated with adenocarcinomas although it may be related to other tumors indicating worse prognosis [2].

Different tumors can cause hypoglycemia. Among tumoral etiologies the insulinoma is the most common cause [3]. Nevertheless, other tumors unrelated to β cell may cause hypoinsulinemic hypoglycemia by producing insulin growth factor-2 (IGF2) or its precursor named as big-IGF2 [3–7]. Doege-Potter syndrome is a paraneoplastic hypoglycemia caused by solitary fibrous tumor [8, 9]. This rare neoplasm can secrete an IGF2 precursor that is a pro-IGF2 or a big-IGF2 [9, 10].

This case report describes a non-diabetic 81-year-old man who presented with Leser-Trélat sign and hypoglycemia secondary to a retroperitoneal malignant solitary fibrous tumor.

## Clinical case

An 81-year-old man with primary hypothyroidism complained about symptoms suggestive of hypoglycemia for 6 months, with progressive worsening, and abdominal pain. He reported rapidly progressive appearance of brownish warty lesions throughout his body, one of them already biopsied (seborrheic keratoses).

**Fig. 1:**
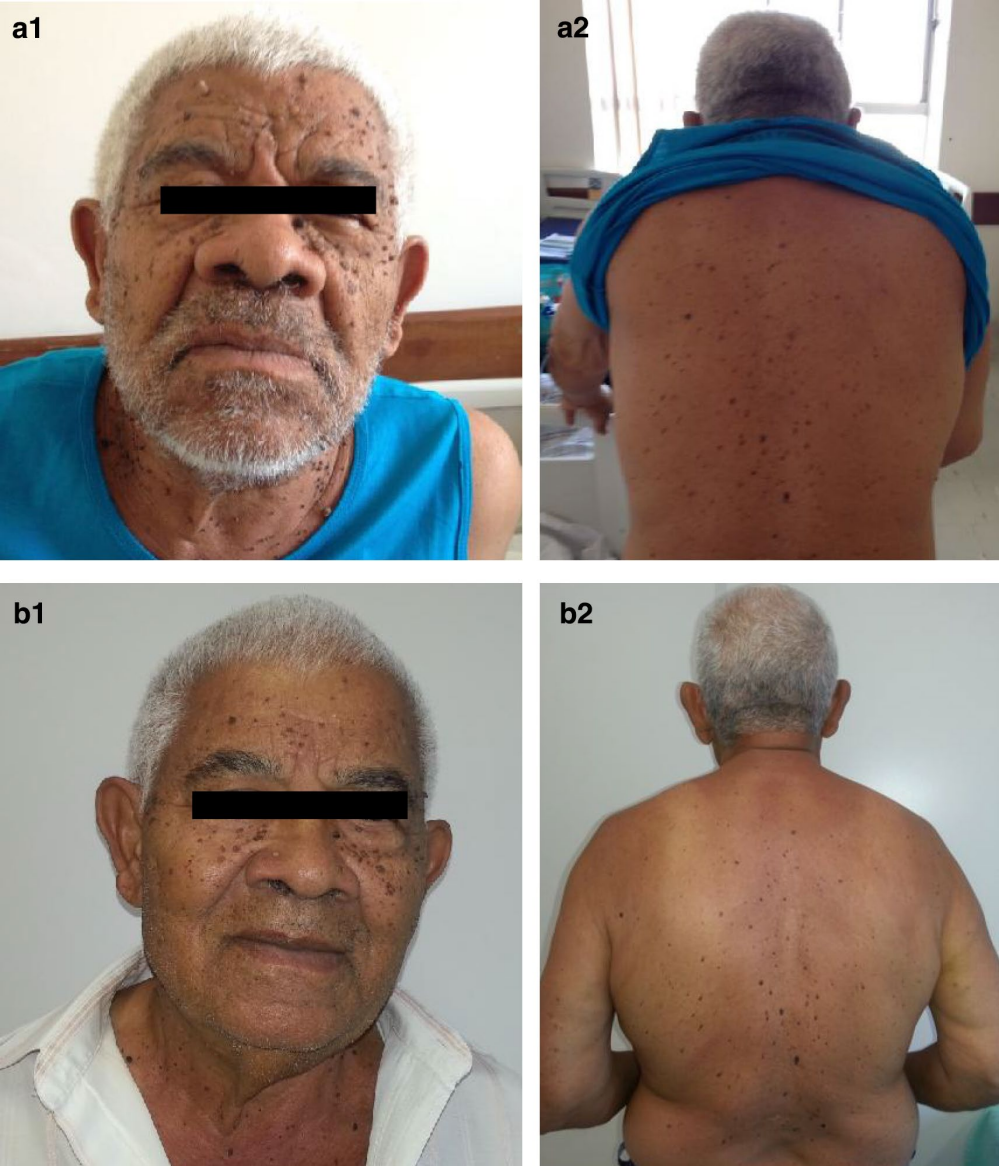
Sign of Leser-Trélat. Multiple lesions on the face (**a**) and back of the patient (**b**) compatible with seborrheic keratoses

He showed, as important, in his physical examination, acromegaloid face, brown warty lesions on the face, trunk and upper limbs (Fig. 1a) and a palpable mass in the right flank. Hematologic, renal and liver function, electrolytes evaluation of his blood and sera were within normal range. The work-up to his hypoglycemia etiology is shown in the Table 1.

**Table 1:** Laboratory tests confirming non-ketotic hypoinsulinemic hypoglycemia by IGF2 production

Test	Result	Cut-off
Morning cortisol (nmol/L)	259.34	171.05–535.24
Potassium (mmol/L)	4.2	3.5–5.0
Glucose (mmol/l)	2.22	3.88–5.49
Thyrotropin (mU/L)	3.07	0.27–4.2
Free T4 thyroxine (nmol/L)	0.013	0.011–0.021
Insulin (pmol/L) in hypoglycemia (2.22 mmol/L)	<1.2	<18
Pro-insulin (pmol/L) in hypoglycemia (2.22 mmol/L)	1.56	<9.4
C Peptide (nmol/L) in hypoglycemia (2.22 mmol/L)	0.032	<0.198
β-hidroxybutyrate (µmol/L)	<9.98	<429.91
Cortisol (nmol/L) in hypoglycemia (1.6 mmol/L)	427.64	>496.62
GH (μg/L)	0.09	≤2.47
IGF-1 (μg/L)	32	55–166
IGF-2 (μg/L)	594	288–736
IGF-2/IGF-1 ratio	18.56	Up to 3

**Fig. 2:**
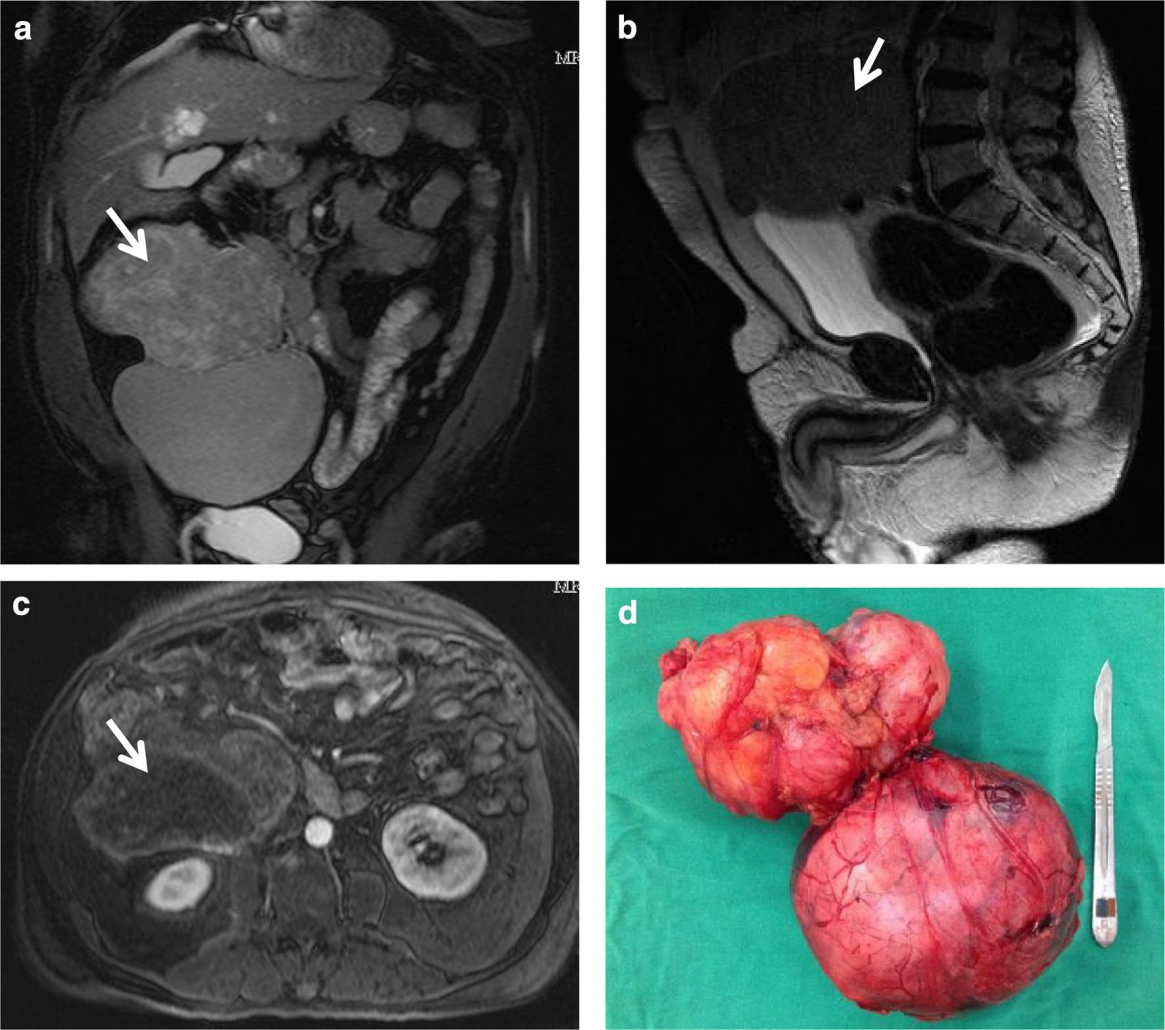
Solitary fibrous tumor from this patient. **a** MRI appearance of the mass in the coronal* plane* shows 18.6 cm in maximal diameter. **b** MRI T2-weighted image in the sagittal* plane* demonstrates the retroperitoneal location of the tumor. **c** contrast-enhanced MRI transverse* plane* indicates a lobulated tumor with solid and cystic componentes. **d** Photograph of the dissected specimen weighing 1.5 kg presents two nodular masses measuring 14.3 and 13 cm with irregular shape and connected by a fibroadipose tissue. There were extensive areas of necrosis

His abdominal magnetic nuclear resonance imaging showed a retroperitoneal expansive mass with 18.6 cm in diameter extremely closed to great abdominal vessels (Fig. 2). Distant metastatic lesions were excluded by tomography screening.

To test the tumor production of IGF2 were collected: growth hormone (GH), IGF1 and IGF2 (radioimmunoassay), confirming the hypothesis (Table 1).

During the 2 months wait period before surgery it was prescribed fractionated diet, oral prednisone (30 mg/day) plus overnight intravenous glucose solution infusion with sporadic episodes of mild hypoglycemia. By the surgery time an encapsulated tumor was resected (Fig. 2). Histopathological and immunohistochemical analysis confirmed malignant solitary fibrous tumor.

The patient showed good health recovery and he was sent to home and to out-patient clinic follow-up without hypoglycemic crises and on oral prednisone treatment that was stopped 6 months later.

By out-patient clinic appointment he referred no hypoglycemic episodes and his acromegaloid features and cutaneous lesions shown an involution (Fig. 1b). He started on local radiotherapy but new tomography screening showed pulmonary nodules suggestive of metastatic disease though he did not present any further hypoglycemia. The patient decided for palliative care and died after 18 months from the diagnosis.

## Discussion

Non-islet cell tumor hypoglycemia should be suspected in any patient with Whipple’s triad and hypoinsulinemic hypoglycemia [4], since several diseases, hormone deficiencies and alcohol intake have been excluded [1, 11]. This patient had a palpable tumor and progressive skin lesions favoring tumoral etiology [3, 4, 7, 12].

The presence of seborrheic keratosis contributed to the suspected of neoplastic syndrome in this patient. This rare situation, [13] due to a tumor, is known as the Leser-Trélat sign. The first description comes from 1956 by Hollander [2, 14]. Seborrheic keratosis usually appears as black or brown warty papules, sharply delineated, with 3 mm mainly on the chest and back. It is a sign of senility but the eruptive and abruptly form can result from inflammatory skin diseases, adverse drug reaction or paraneoplastic syndrome [14, 15]. Lesions appear mainly on the chest and back, bigger than the usual and the pre-existing lesions can increase in size [12].

It is more frequent (68 %) that the skin condition precedes the diagnosis of the tumor, [12, 13, 16] and it is resulted of the release of tumor growth factors [12]. The most frequent tumors involved are adenocarcinomas, but other tumors have been described [2, 16]. The best treatment is the tumor resection, but the skin response is variable [13]. Although the exactly pathogenesis is uncertain it involves the cytokines produced by the neoplastic cells as IGF1 and epidermal growth factor that act in the surface of epidermal cells [16].

In half of the patients with non-islet cell tumor hypoglycemia, hypoglycemia comes after the tumor has been found [5] and fasting hypoglycemia and neuroglycopenic symptoms are more common. This tumor is most commonly diagnosed in the fifth or sixth decades of life and symptoms can be present for weeks or months before it [1, 3, 4, 6, 7, 12]. Weight loss, palpable mass and pain are the main clinical manifestations. In rare patients there is acromegaloid features [3, 7]. It was first described in 1929 but only in 1988 it was associated with IGF2 production [3, 7, 17, 18].

Usually, 80 % of the circulating mature IGF2 stays inactive bounded to insulin like growth factor binding protein 3 (IGFBP3) and to acid labile subunit [7, 19–21]. In patients with non-islet cell tumor the pool of big-IGF2 increases and 80 % of this molecule is bounded to IGFBP3 only, forming a lower molecular weight that crosses the endothelial barrier and acts on insulin receptors [4, 7, 20, 22].

Increased glucose uptake, lower insulin and glucagon secretion, decreased lipolysis and liver gluconeogenesis are some effects of the massive secretion of IGF2 that lead to hypoglycemia [3, 5]. Autocrine and paracrine action also promotes tumor growth. The big-IGF2 suppresses GH secretion and it causes synthesis reduction of IGF1, IGFBP2 [1, 3, 18, 20] increasing the bioavailability of IGF2 [1, 3, 4, 6, 20, 22].

Laboratorial tests can show normal level of IGF2 but elevated ratio of IGF2/IGF1. A ratio up to three is considered normal; if it is higher than ten, in the presence of non-ketotic hypoglycemia and suppressed GH, it is reported to be virtually pathognomonic of non-islet cell tumor hypoglycemia [7, 9, 11]. Measurement of free IGF2 or its precursors are not widely available but it is known that free IGF2 and the ratio pro-IGF2/IGF1 are increased [1, 3, 4, 9]. The presence of lower levels of GH and IGF1 and a positive response to glucagon-stimulation test can support the diagnosis when the IGF2 assays are unavailable [4].

The tumors are usually large and well differentiated, with epithelial or mesenchymal origin, slow growing, arising in thorax, pelvis, or retroperitoneum [1, 3, 6]. The most common epithelial tumor is hepatocellular carcinoma and the mesenchymal are fibrosarcomas and solitary fibrous tumor [3, 5, 7, 10, 23]. The latter is a rare neoplasm, with a widespread distribution, involving pleura, lungs, pelvic and abdominal organs or retroperitoneum [9, 10, 23]. It affects men and women equally, 50 % of then were asymptomatic and the majority were found as incidentalomas on diagnosis. The most symptomatic tumors have extrathoracic location and mass effects (urinary retention, venous thrombosis and abdominal pain) [23].

Solitary fibrous tumor usually follow a benign clinical course although progress with distant metastasis or local recurrence may arise in some cases of large tumors. Extra thoracic location, paraneoplastic hypoglycemia, positive margins, high cellularity and mitotic activity, necrosis and nuclear pleomorphism are predictors of worse clinical outcome [10, 23].

Hypoglycemia due to a solitary fibrous tumor was first reported by Doege and Potter in 1929. Since then, this finding is called Doege-Potter Syndrome but is very rare (less than 5 % of all solitary fibrous tumors) [8–10]. Among 79 cases of solitary fibrous tumors reported by Jason et al. only two presented hypoglycemia [23].

Regardless of histology, surgical resection is the therapy of choice. Complete surgical resection solves hypoglycemia [3, 7, 9, 24] and reverses those cases of acromegaloid fenotype [4]. When total resection is impossible the debulking technic can be used to ameliorate hypoglycemia or totally solve it in some cases [7]. Adjuvant therapy like tumor embolization, radiotherapy or chemotherapy may bring success in other cases [3, 7].

Short- term measures for avoiding hypoglycemia include corticoid, glucagon, diazoxide and glucose intake. When hypoglycemia persists after surgical intervention, multiple medical modalities have been employed. Although many of these tumors have somatostatin receptors, the use of analogs was not effective in controlling hypoglycemia and somatostatin analogue scintigraphy could not predict treatment response [3].

There are many reports of successful treatment with recombinant growth hormone, although it does not normalize IGF2 levels. Nevertheless, this therapy is expensive, requires high doses, and has many adverse effects [4, 12]. It’s use should be avoided when there are acromegaloid features [4].

The best clinical treatment option is the use of glucocorticoids with immediate effect on hypoglycemia in doses equivalent to 30–60 mg/day of prednisone [3, 4]. Moderate to high doses also could promote tumor reduction [7]. It is the only medical treatment that can suppress tumor production of IGF2, leading to normal levels of C-peptide, insulin and glucose [7, 14].

According to the English literature review, Leser-Trélat sign and hypoinsulinemic hypoglycemia by a solitary fibrous tumor is extremely rare. Whipple’s triad and hypoinsulinemic hypoglycemia, with low levels of GH and IGF1 suggest the diagnosis and the increase of IGF2/IGF1 rate confirms. Some weeks after the surgery the patient showed remission of hypoglycemia without glucocorticoids use and improvement of seborrheic keratosis and acromegaloid features.
